# Is obsessive–compulsive personality disorder related to stress‐related exhaustion?

**DOI:** 10.1002/brb3.2171

**Published:** 2021-05-10

**Authors:** Susanne Gulin, Susanne Ellbin, Ingibjörg H. Jonsdottir, Ann‐Sophie Lindqvist Bagge

**Affiliations:** ^1^ The Institute of Stress Medicine Region Västra Götaland Gothenburg Sweden; ^2^ School of Public Health and Community Medicine Institute of Medicine Gothenburg University Gothenburg Sweden; ^3^ Department of Psychology Gothenburg University Gothenburg Sweden

**Keywords:** exhaustion disorder, ED, obsessive**–**compulsive personality disorder, OCPD, Perfectionism

## Abstract

**Objective:**

Recovery from stress‐related diagnoses can, in some cases, be long‐lasting, and several different factors could be related to such a lengthy recovery. One plausible aspect is obsessive**–**compulsive personality disorder (OCPD), which has previously been seen to be related to stress‐related mental health. Thus, the aim of this study was to investigate whether recovery from exhaustion disorder (ED) is associated with OCPD.

**Methods:**

This study includes data from 147 patients (78% women, mean age 52.4 ± 9.8 years) who have been treated for ED. Clinical assessment was performed 7–10 years after first seeking care identifying patients with residual exhaustion. Symptoms of OCPD were concomitantly measured and several aspects of work‐ and private‐related stress exposure.

**Results:**

The main result of this study is that patients with residual clinical ED report OCPD to a greater extent, compared with patients who no longer fulfill the clinical criteria for ED, 7–10 years after seeking care. Patients with OCPD that have not recovered report “excessive devotion to work” to a higher degree than patients with OCPD that have recovered.

**Conclusion:**

The results indicate that factors related to OCPD may be of clinical importance for the patient's recovery from ED. However, prospective studies should be conducted and studies elucidating whether symptoms of exhaustion among patients with OCPD can be affected by therapeutic interventions.

## Graphical abstract

Recovery from stress‐related diagnoses can, in some cases, be long‐lasting and several different factors could be related to such a lengthy recovery. The aim of this study was to investigate whether recovery from exhaustion disorder (ED) is associated with OCPD in 147 former patients with ED. The main result is that patients with residual clinical ED report OCPD to a greater extent compared with patients who do not longer fulfill the clinical criteria for ED, 7‐10 years after seeking care. Patients with OCPD that have not recovered report “excessive devotion to work” to a higher degree than patients with OCPD that have recovered.

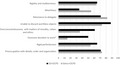

## 1 INTRODUCTION

Stress‐related diagnoses have since 2010 been the fastest‐growing cause of sick leave in Sweden according to the Swedish Social Insurance Agency (Försäkringskassan, 2017b). In 2016, psychiatric diagnosis, especially reaction to severe stress and adjustment disorders (F43) (World Health Organization, 1992), was the most common cause (44%) for long‐term sick leave in Sweden (Försäkringskassan, 2017, 2017). A recent review of burnout in workplaces indicates that increased sick leave due to stress at work is a general trend seen across Europe (Burnout in the workplace: A review of data and policy responses in the EU 2018), and it is well known that poor work environment is strongly related to stress‐related mental health problems (Harvey et al., 2017).

In Sweden, the clinical diagnosis *Exhaustion Disorder* (ED) is used for more severe stress‐related illness, internationally referred to as clinical burnout (Grossi et al., 2015). The diagnose was in 2005 incorporated in the Swedish version of the ICD‐10 with the diagnostic code *Other* *reactions to severe stress* (F43.8) (World Health Organization, 1992). Exhaustion disorder defines patients with exhaustion that has developed because of identifiable stressor(s) that have been present for at least six months (Table 1). The symptoms of ED and burnout are closely related, and it has previously been shown that patients fulfilling the diagnostic criteria for ED can also be described as clinically burned‐out (Glise et al., 2012; Jonsdottir et al., 2009). Thus, there is a clear overlap between the concept of the psychological construct burnout and the clinical diagnosis ED. However, the concept of burnout cannot be used in clinical practice and as early as the mid‐1980s it was argued that a distinction should be made between burnout as a work‐related stress syndrome and burnout as a clinical mental disability (Paine, 1982; Schaufeli & Greenglass, 2001). Attempts have been made to adapt the burnout concept to be more usable in clinical practice but is has been found that the most utilized burnout tool, the Maslach Burnout Inventory, does not seem to be suitable as a diagnostic tool for patients (Kleijweg et al., 2013). Furthermore, the burnout concept expects that the problems are work‐related but clinical patients seeking care for exhaustion report both private‐related and work‐related stress exposures as contributing factors to their exhaustion (Hasselberg et al., 2014).

**TABLE 1:** Diagnostic criteria for Exhaustion Disorder according to the National Board of Health and Welfare (2003)

A	Physical and mental symptoms of exhaustion with minimum two weeks duration. The symptoms have developed in response to one or more identifiable stressors which have been present for at least 6 months.
B	Markedly reduced mental energy, which is manifested by reduced initiative, lack of endurance, or increase of time needed for recovery after mental efforts.
C	At least four of the following symptoms have been present most of the day, nearly every day, during the same 2‐week period:
1	*Persistent complaints of impaired memory*.
2	*Markedly reduced capacity to tolerate demands or to work under time pressure*.
3	*Emotional instability or irritability*.
4	*Insomnia or hypersomnia*.
5	*Persistent complaints of physical weakness or fatigue*.
6	*Physical symptoms such as muscular pain, chest pain, palpitations, gastrointestinal problems, vertigo or increased sensitivity to sounds*.
D	The symptoms cause clinically significant distress or impairment in social, occupational, or other important areas of functioning.
E	The symptoms are not due to the direct physiological effects of a substance (e.g., a drug of abuse, a medication) or a general medical condition (e.g., hypothyroidism, diabetes, infectious disease).
F	If criteria for major depressive disorder, dysthymic disorder, or generalized anxiety disorder are met, exhaustion disorder is set a comorbid condition.

Several studies have shown that recovery from ED could be long‐lasting. For example, as many as 37% of patients did not show any recovery regarding symptoms of burnout despite a year of rehabilitation and an additional six months of therapeutic follow‐up (Hatinen et al., 2009). Similar results were seen in a study from our research group, showing that one‐third of patients still report symptoms of burnout, which was not related to sex or age, following 18 months of treatment (Glise et al., 2012). Longer follow‐up has shown that as many as two‐thirds of clinical burnout patients reported mental fatigue and physical fatigue three years after seeking care (Stenlund et al., 2012). A recently published study from our research group revealed that as many as one‐third of patients are clinically diagnosed with ED seven years after seeking professional care for their ED symptoms (Glise et al., 2020). The observed long‐lasting recovery for some individuals with ED, calls for further studies focusing on plausible mechanisms that could explain the persistent and residual ED symptoms.

One factor associated with ED is personality trait related to compulsive overworking, which is described as an irresistible inner drive to work excessively hard (Schaufeli et al., 2009). Another personality trait associated with ED is perfectionism (Hill & Curran, 2016). Perfectionism is defined as a multidimensional concept including high personal standards and demands in combination with overly self‐critical evaluation (Frost et al., 1990). Hallsten and colleagues (Hallsten et al., 2005) showed that people with stress‐related mental illness are often described as perfectionist with high internal performance demands. Van Yperen and colleagues (Van Yperen et al., 2011) found perfectionism to be a common factor for clinical burnout, anxiety, and depression. The relationship between perfectionism and stress/burnout has been observed and discussed in several different populations (Atienza et al., 2020; Collin et al., 2020; DeCouto et al., 2020; Hassmen et al., 2020; Rice & Liu, 2020). Perfectionism has been shown to be a risk factor for burnout through the psychosocial load created by constant concern about making mistakes or not live up to own unreasonably high standards (Hill & Curran, 2016; Osenk et al., 2020). Unrealistically, high personal standards and self‐criticism have also been suggested to be associated with anxiety and depression (Antony & Swinson, 2009). High‐performance demands in today's working life might also contribute to that these individuals perceive that they feel that they have to perform at standards exceeding what can be considered healthy for the individual (Harenstam, A., & Group, M. O. A. R, 2005; Kashefi, 2009). Bovornusvakool and colleagues (2012) have identified perfectionism as being a key factor in the development of compulsive overwork (Bovornusvakool et al., 2012). Perfectionism including difficulties in dealing with high demands has also been reported to be one of several barriers in the return to recovery process in patients with common mental disorder, which include stress‐related mental health problems (Noordik et al., 2011; Pasquini et al., 2015). Perfectionism and excessive devotion to work are aspects included in the Structured Clinical Interview for DSM‐IV Axis‐II (SCID (First et al., 1999) when evaluating obsessive–compulsive personality disorder (OCPD). The OCPD has further been described as having a strong relationship to the exhaustion aspect in burnout (Rossler et al., 2015).

Since perfectionism has been shown to be associated with burnout the present study focuses on OCPD, since—unlike obsessive–compulsive disorder (OCD) ‐ OCPD includes perfectionism in its diagnostic criteria (Van Yperen et al., 2011). OCPD is a cluster C personality disorder characterized by excessive need for orderliness, neatness, and perfectionism (Diagnostic and statistical manual of mental disorders (2013)).

The main aim of the present study was thus to investigate whether obsessive–compulsive personality disorder is related to the recovery of stress‐related exhaustion disorder.

More specifically, the primary aim is to explore whether the prevalence of self‐reported OCPD differ between patients that seven to ten years after being diagnosed still fulfill the clinical criteria for ED compared with patients who do not longer fulfill the clinical criteria for ED. Secondly, to explore whether OCPD symptoms differ between patients with OCPD who have recovered from ED compared with patients with OCPD that have not recovered from ED. And thirdly, to explore whether contributing exposure factors at work and/or in private life measured both at the onset of the disease and at seven years follow‐up differ between patients with OCPD who have recovered from ED compared with patients with OCPD that have not recovered from ED.

## 2 METHODS

### 2.1 Participants and procedure

This study is part of a longitudinal study conducted at the Institute of Stress Medicine (ISM), a specialist outpatient clinic for patients with ED in Gothenburg, Sweden. The patients included in this study participated in a clinical assessment, 7–10 years after seeking care. Initially, the patients were referred to ISM from primary care units or occupational healthcare centers. Criteria for being accepted as a patient at ISM were that the patient fulfilled the criteria for ED (Appendix 1) and had been on sick leave for a maximum of six months. When setting the ED diagnosis, structured clinical interview were conducted including differential diagnostic procedures excluding patients with generalized pain, fibromyalgia, chronic fatigue syndrome/myalgic encephalomyelitis, thyroid disease, vitamin B‐12 deficiency, obesity, alcohol / drug addiction, other psychiatric illness than depression and anxiety, and other somatic disease that could explain fatigue. The mean time for treatment at the ISM was approximately 18 months, and the treatment has previously been described in detail (Glise et al., 2012).

**FIGURE 1:**
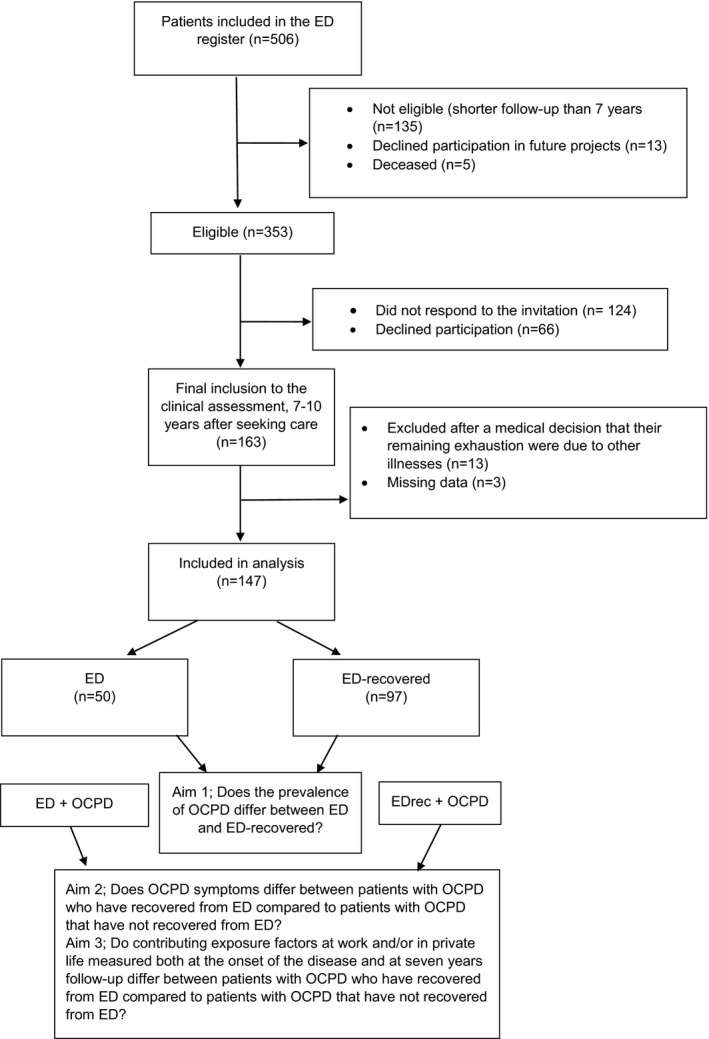
Flow chart of the total of *N* = 506 patients included in the exhaustion disorder (ED) patient register, showing that 334 patients had passed 7 years follow‐up. Among them, 163 patients accepted to participate in a clinical assessment, 7–12 years after seeking care. Thirteen patients were excluded since they were judged to be exhausted due to other clinically reasons than stress exposure. Three patients had missing data on the primary outcome measure in this study, that is, Structured Clinical Interview for DSM‐IV Axis‐II Personality Questionnaire (SCID‐II Personality Form). A final number of 147 patients were thus included in this study, of which 50 are still considered exhausted (ED), and 97 do not longer fulfill the clinical criteria for exhaustion disorder (EDrec). In the first aim, these two groups (ED and EDrec) are compared. When analyzing the second and the third aim the ED and EDrec groups are compared but only including those that fulfill the criteria for OCPD

Following treatment at the clinic, the patients were invited to participate in several follow‐ups (2, 3, 5, 7, and 10 years). All patients that had passed seven years follow‐up (353 out of 506 patients) were asked to participate in a clinical assessment (Figure 1 for flow chart) involving a doctor's visit to further assess residual stress‐related exhaustion and completion of a number of assessment scales, including the Structured Clinical Interview for DSM‐IV Axis‐II Personality Questionnaire (SCID‐II Personality Form). The seven‐year limit was chosen on the basis that our clinical experience has shown that many patients have long‐term symptoms, often several years after completion of treatment. Of the 353 patients, 163 patients (46%) accepted to participate but 13 patients were excluded since they were judged to have exhaustion due to other reasons than stress exposure. The procedures for the assessment have been previously described (Glise et al., 2020). Additional three patients were excluded because of incomplete questionnaires of central importance for this study. Thus, 147 patients were finally included in this current study (50 still reporting ED (ED)) and 97 no longer fulfilling the clinical criteria at seven years follow‐up (EDrec). Majority or 78% are women and average age of the patients was 52.4 ± 9.8 years (range 28 – 74 years).

Drop‐out analysis comparing nonparticipant but eligible (*n* = 206) with participants (*n* = 147) shows that significant larger proportion of women agreed to participate (78% woman versus 67% men (*p* = .03)) and that mean age was higher for the participants (44 years (*SD* 9.66)) compared with nonparticipants (41 years (*SD* 8.99) *p* = .006)). Educational level and level of symptoms of burnout, depression and anxiety measured at both baseline and follow‐up, did not differ between participant and nonparticipant (data not shown).

### 2.2 Instruments

*Structured Clinical Interview for DSM‐IV Axis‐II Personality Questionnaire (SCID‐II Personality Form)* is a self‐assessment form used as a screening tool for all personality syndromes in DSM‐5 (First et al., 1999). The personality form contains 117 questions that are answered with either yes or no. The personality form is intended to be used as a screening tool for a diagnostic interview according to SCID‐II and risks becoming over‐inclusive if used separately (First et al., 1999). Since the questionnaire was designed as a screening instrument preceding a clinical interview, it could, therefore, be expected to be over‐inclusive with high sensitivity and low specificity (First et al., 1999). Studies have been conducted where the SCID‐II‐Qs properties as a standalone instrument was tested, showing that this is feasible, however, with the adjustment that the cut‐off for each personality disorder is adjusted with one point in order for the SCID‐II‐Q to be used as a screening device in epidemiological studies (Ekselius et al., 1994). For OCPD, the adjusted threshold is 5 points (Ekselius et al., 1994). In this study, definition of OCPD was based on the five‐point limit on the SCID‐II personality form where scores ≥5 were categorized as OCPD and <5 points were categorized as no‐OCPD.

### 2.3 Measurements of stressors contributing to the exhaustion

In conjunction to the clinical assessment, seven years or more after first seeking care, the patients were asked to fill in a questionnaire including a list of different work‐related and private‐related stressors (Appendix 1). Thus, the patients were asked to state whether the stressor or the particular situation was judged by him or her to contribute to their exhaustion when they initially sought care (T1) and/or whether the stressor or situation was judged to be a considerable strain for the individual as to today, that is, 7 years or more after initially seeking care (T2). The stressors listed is a result of a content analysis of the patient´s medical records journals previously described by Hasselberg et al (Hasselberg et al., 2014). Briefly, during the clinical interview when the patient's first visit the clinic, stressors were identified by the physician together with the patient. This was a part of the diagnostic procedure, and the information obtained from this content analysis formed the basis for the questionnaire used in this study. The content analysis resulted in a total of 24 categories of stressors, of which 11 were related to work, and 13 were nonwork related (Hasselberg et al., 2014). Thus, these 24 items are listed and two additional items (high internal demands and existential worries) that were added since these appeared in a later content analysis only including younger patients (<30 years) seeking for exhaustion disorder, resulting in a total of 26 items (Appendix 1). An open alternative was also included given the possibility of adding exposure not already listed.

*Hospital Anxiety and Depression Scale* *(HAD)* is a self‐assessment scale for anxiety and depression (Zigmond & Snaith, 1983). HAD consists of two subscales, HAD anxiety and HAD depression, each with seven statements that are answered on a four‐grade Likert scale from 0 to 3. The item scores are summarized within each subscale, with scores ranging from 0 to 21 points. Scores above 10 indicate anxiety / depression of clinical significance. A literature review by Bjelland, Dahl, Haug, and Neckelmann (Bjelland et al., 2002) showed good discriminatory validity and good internal consistency for HAD. A Swedish evaluation of HAD by Lisspers, Nygren, and Söderman (Lisspers et al., 1997) assessed the self‐assessment scale as a useful indicator of clinical depression and anxiety.

### 2.4 Data analysis

Descriptive statistics were given in percentages and counts for categorical variables and means and standard deviations (*SD*) for continuous variables. Pearson's chi‐square test and *t* test were used to test for significant differences for categorical and continuous variables, respectively. Pearson's chi‐square test was used to analyze whether the prevalence of OCPD differed between the groups. The mean differences between the percent of patients in each group (ED+OCPD and EDrec+OCPD) reporting each stressor was calculated. Due to small sample size of patient with OCPD (17 in the ED group and 11 in the EDrec group), all stressors initially reported by ≤10 patients (total for the ED and EDrec) were excluded from the analysis. These were job insecurity, traumatic event at work, poor physical work environment, irregular working hours, separation, change in family composition, worries about one's health, worries about personal injury or illness, financial worries, residential stressor, voluntary engagement, legal matter, loneliness, death of a family member, caring for a family member, and being a single parent. Confidence intervals for differences in proportions between the groups are calculated using ExactCIdiff package in R. All other analyses were done with a two‐sided significance level of *p* <.05. For the statistical data analyses, SPSS Statistics 25 was used.

### 2.5 Ethical approval

The study was approved by the Regional ethical review board in Gothenburg Sweden 2015–10–16, reference number 668–15. All participants gave their written informed consent before entering the study.

## 3 RESULTS

There were no significant differences between ED and EDrec regarding age, sex, or follow‐up time, that is, duration in years between baseline and follow‐up. Significantly, higher percent of the patients in the ED group scored above 10 on HAD anxiety (78%) compared with EDrec (54%) at baseline (*p* = .002), whereas HAD depression score did not differ between the groups (Table 2). At follow‐up, the group that were clinically judged to have ED also reported significantly higher scores on symptoms of both depression and anxiety (Table 2).

**TABLE 2:** Baseline characteristics of the patients included in this study. Firstly, the group of patients that still fulfill the clinical criteria for exhaustion (ED) are compared with those no longer fulfilling the clinical criteria for exhaustion (EDrec) at follow‐up. The second comparison is between the patients reporting OCPD and still reporting ED (ED+OCPD) compared with patients with OCPD that have recovered (EDrec+OCPD)

	ED *N* = 50	EDrec *N* = 97	ED +OCPD *N* = 17	EDrec +OCPD *N* = 11	ED versus EDrec *p*	ED +OCPD versus EDrec +OCPD *p*
Sex‐Female/male (%)	78%/22%	77%/23%	77%/23%	64%/36%	0.925	0.463
Age mean (*SD*)	43 (9.5)	44 (9.8)	42 (10.2)	40 (10.8)	0.656	0.582
Education in years (*SD*)	9.5 (1.3)	9.7 (0.9)	14.6 (3.2)	13.6 (2.3)	0.115	0.279
Mean time (years) from baseline to follow‐up (*SD*)	9.1 (1.6)	9.4 (1.6)	8.8 (1.5)	9.1 (2.0)	0.917	0.713
HADS depression scale >10 *n* (%) at baseline	20 (41%)	31 (32%)	6 (35%)	5 (46%)	0.309	0.591
HADS anxiety scale >10 *n* (%) at baseline	39 (78%)	51 (54%)	14 (82%)	7 (63%)	0.002	0.264
HADS depression scale >10 *n* (%) at follow‐up	10 (20%)	1 (1%)	6 (35%)	1 (9%)	>0.001	0.118
HADS anxiety scale >10 *n* (%) at follow‐up	15 (30%)	8 (8%)	5 (29%)	3 (27%)	0.001	0.903

Abbreviations: ED, exhaustion disorder (i.e., still fulfilling criteria for ED at the follow‐up 7–12 after ED baseline diagnosis; EDrec, clinically recovered from exhaustion disorder (i.e., no longer fulfilling criteria for ED at the follow‐up); OCPD, obsessive**–**compulsive personality disorder, HADS, Hospital and anxiety depression scale, SD, standard deviation.

### 3.1 Aim 1: Prevalence of OCPD in (ED versus ED‐rec) patients

Patients clinically judged to still have ED after 7–10 years (ED (*n* = 50)) were compared with patients that were clinically judged to be recovered according to the criteria (ED‐rec (*n* = 97)) regarding OCPD. Significantly, higher percent of patients in the ED group, 34% (*n* = 17) met the OCPD criteria according to SCID (referred to as ED+OCPD) as compared to 12% (*n* = 11) in the EDrec group (referred to as EDrec+OCPD) (*p* = .001). No significant difference was seen between ED+OCPD group and EDrec+OCPD group regarding symptoms of depression and anxiety, either at baseline or at follow‐up (Table 2).

### 3.2 Aim 2: OCPD symptoms in patients with OCPD who have recovered (EDrec+OCPD) compared with those who have not recovered (ED+OCPD)

**FIGURE 2:**
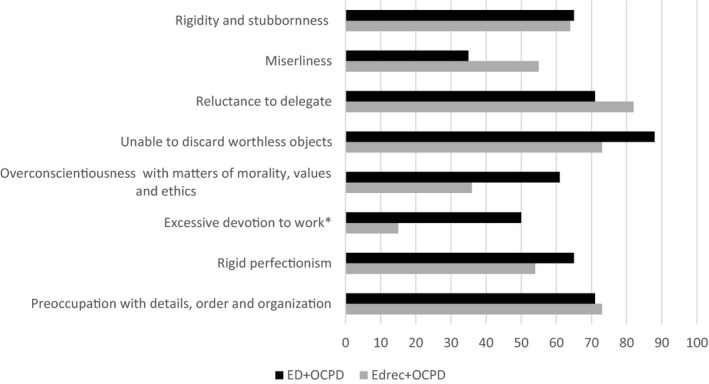
The percent of patients scoring above cut‐off for each OCPD symptom included in the Diagnostic criteria for OCPD according to DSM‐IV. No difference was seen between the group still clinically exhausted (ED+OCPD) compared with those who have recovered (EDrec+OCPD) except for the symptom “excessive devotion to work” reported by significantly more patients that are still exhausted

The pattern of OCPD symptoms in patients reporting OCPD is similar regardless if they have recovered (EDrec+OCPD) or are still clinically exhausted (ED+OCPD), 7–10 years after seeking care. The only significant difference seen was that as many as 50% of those still clinically exhausted (ED+OCPD) score above cut‐off for the symptom “excessive devotion to work” compared to 15% among those who have recovered (EDrec+OCPDfigure) (*p* =.031) (Figure 2).

### 3.3 Aim 3: The character of work‐ and private‐related exposure in patients with OCPD who have recovered (EDrec+OCPD) compared with those who have not recovered (ED+OCPD)

**FIGURE 3:**
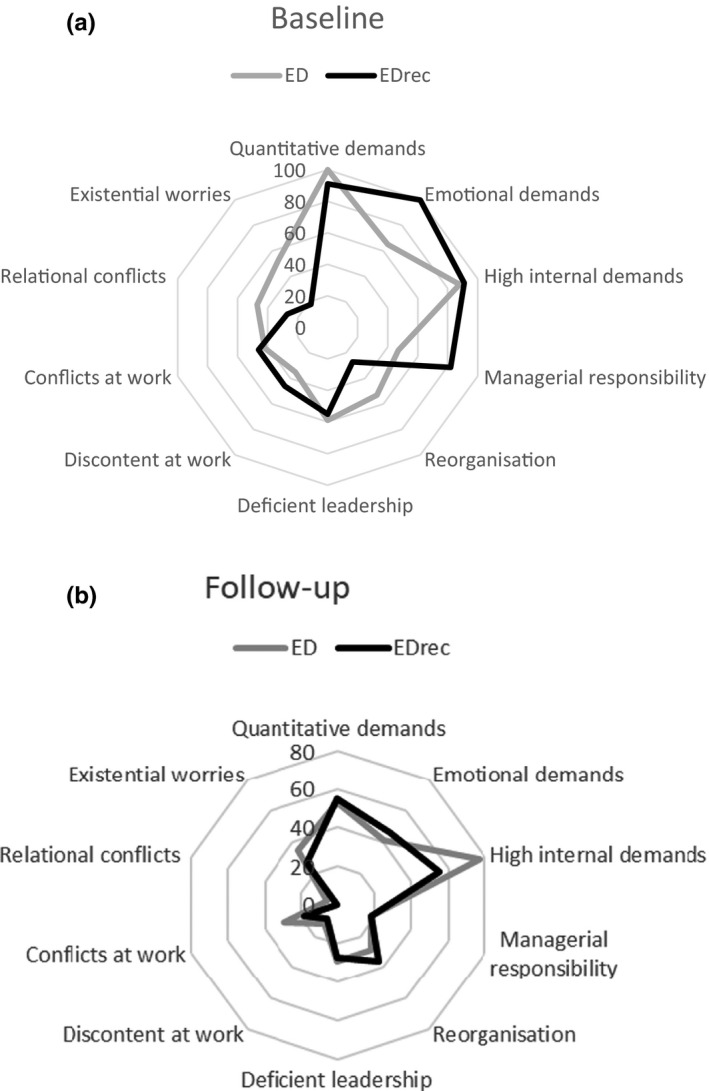
Radar charts showing the percentages of patients in each group (ED+OCPD and EDrec+OCPD) that report the respective exposure factor as a contributing factor to their exhaustion when they first sought care (a) and/or that the exposure factor were considered to be a causing strain in their life at seven years follow‐up (b)

The patients were asked to fill in a questionnaire asking about self‐reported stress exposure that was judged by the patients to be a contributor to their exhaustion at the baseline (T1) and at 7–10 years follow‐up (T2). Only, stressors reported by more than 10 patients at baseline were included in the analysis. Differences in percentages and 95% CI are shown in Table 3, noticing the rather large CI, mostly due to small sample size. The only significance was seen for emotional demands at baseline reported by all patients in the EDrec+OCPD (100%) compared to 65% among those still exhausted (ED+OCPD). Looking at the pattern for all stressors included, both groups show a considerable change toward less exposure at follow‐up compared with baseline (Figure 3a,b).

**TABLE 3:** Self‐reported exposure factors (stressors) reported to contribute to the exhaustion when the patients first sought care (T1) and at 7–10 years follow‐up (T2). The percentage of patients reporting the particular stressor in each group is compared for each time point. The groups compared are patients that report OCPD and still fulfill the criteria for Exhaustion disorder (ED+OCPD) 7–10 years after seeking care and the group of patients that report OCPD but have clinically recovered from ED (EDrec+OCPD)

	Time	ED+OCPD % (*n*)	EDrec+OCPD % (*n*)	Differences in percentages (95% CI)
Quantitative Demands	T1	100 (17)	91 (10)	9.1 (−11.1;41.3)
	T2	53 (9)	55 (6)	−1.6 (−39.1;36.4)
Emotional Demands	T1	65 (11)	100 (11)	−35 (−61.6;−5.3)
	T2	41 (7)	46 (5)	−4.3 (−41.5;32.9)
High internal demands	T1	88 (15)	91 (10)	−2.7 (−27.4;28.1)
	T2	77 (13)	55 (6)	21.9 (−15.4;56.9)
Managerial responsibilities	T1	47 (8)	82 (9)	−34.8 (−65.7;5.2)
	T2	18 (3)	18 (2)	0 (−35.8;31.1)
Reorganization	T1	53 (9)	27 (3)	25.7 (−14.8;58.0)
	T2	29 (5)	36 (4)	−7.0 (−43.4;30.0)
Deficient Leadership	T1	59 (10)	55 (6)	4.2 (−32.9;41.5)
	T2	29 (5)	27 (3)	2.1 (−34.8;37.6)
Discontent at work	T1	35 (6)	46 (5)	−10.1 (−46.9;27.1)
	T2	12 (2)	9 (1)	2.6 (−28.1;27.4)
Conflicts at work	T1	42 (7)	46 (5)	−4.3 (−41.5;32.9)
	T2	29 (5)	18 (2)	11.2 (−25.9;43.9)
Relational conflicts in private live	T1	47 (8)	27 (3)	19.8 (−18.9;54.1)
	T2	6 (1)	0 (0)	5.9 (−20.3;29.2)
Existential worries	T1	53 (9)	18 (2)	34.8 (−5.2;65.7)
	T2	35 (6)	27 (3)	8.0 (−29.6;40.3)

## 4 DISCUSSION

The main result of this study is that patients with residual clinical ED report OCPD to a greater extent compared with patients who do not longer fulfill the clinical criteria for ED, 7–10 years after seeking care. The pattern of OCPD symptoms is similar for patients reporting OCPD regardless if they have recovered from ED or not. The only exception is “excessive devotion to work,” which was significantly more common among the patients still reporting exhaustion. Both groups report similar exposure pattern at baseline and follow‐up with a clear decrease in both work‐related and private‐related exposure, indicating that the patients have made several positive changes compared with their initial situation.

Thus, higher proportion of patients still clinically exhausted report OCPD symptoms, indicating that factors included in OCPD such as perfectionism, rigidity, and excessive devotion to work seem to be one of several plausible factors related to poor recovery in some patients. However, it might not be these characteristics as such that are problematic. Thus, the complexity of the interaction between individual and organizational factors needs to be addressed, particularly regarding excessive devotion to work. It is well known that poor organizational and psychosocial work environment, including heavy workload, poor control, lack of support and autonomy are clearly related to stress‐related exhaustion and burnout and the foremost action regarding prevention should be aimed toward improving the psychosocial work situation for the employees (Harvey et al., 2017).

Thus, high‐performance requirements at many work places can lead to difficulties in limiting the workload and meeting own needs, which often means that physical and mental limits are exceeded (Blom, 2012). Indeed, the OCPD symptom “excessive devotion to work” was reported by far more patients in the group that had not recovered compared with those reporting OCPD that are judged to be clinically recovered. Excessive devotion to work (cf. compulsive overworking, workaholism) has been described as a somewhat persistent behavioral pattern (Atroszko et al., 2020). An individual's self‐imposed workload might partly be modified from work‐organizational interventions, but also needs to be addressed from the individual's perspective by clarifying the driving forces behind this behavior.

Atroszko and coworkers (Atroszko et al., 2020) recently published a model explaining the relationship between individual vulnerabilities (e.g., rigid perfectionism), organizational factors at work such as job demand, the macro‐level including state policies and cultural values and health outcome such as burnout. The authors point out that all levels are connected and need to be considered when working with workplaces regarding prevention of stress‐related problems. A similar process is described in the effort‐reward model, where imbalance between effort and reward leads to over‐commitment by some people, that is, they increase the efforts even more in the hope of getting appreciation, which makes them extra vulnerable to stress and exhaustion in a psychosocially stressful work environment (Siegrist, 2005).

Related to the role of culture in one's approach to work, few studies have de facto examined the influence of cultural changes on perfectionism (Curran & Hill, 2019; DeCouto et al., 2020). The study by Curran et al. (2019) found an increase in perfectionism that coincided with cultural changes over the last three decades in Canada, the United States, and Great Britain (Curran & Hill, 2019). It can be concluded that culture has an impact on perfectionism. Though the present result is based in a Swedish context, it is believed that the results are generalizable to work‐related attitudes within Western cultures.

Another important aspect to raise is the self‐criticism created by an experienced discrepancy between performance, the own high rigid standards, and the self‐worth based on achievements and an endeavor to fulfill their own unreasonably high standards regardless of consequences. This has been described in previous studies as clinical perfectionism (Shafran et al., 2002), performance‐based self‐esteem (Hallsten et al., 2005), and conditional self‐esteem (Blom, 2012). The lack of adaptation combined with poor work environment and excessive demands thus increases the perceived discrepancy, which can lead to self‐criticism and stressful situations including excessive workload that is impossible to deal with when suffering from exhaustion.

Summarizing, the maintenance of situation related to excessive workload, poor psychosocial work environment, high internal demands, and self‐criticism could plausibly result in preservation of symptoms of exhaustion over time in a subgroup of patients with ED as seen in this study. Whether behavioral pattern associated with OCPD symptoms is a pre‐morbid feature contributing to the exhaustion or if these behavioral strategies are developed because of the exhaustion cannot be answered with this cross‐sectional data.

Although not significant, due to small sample size, we also notice that a majority of the group that have not recovered, or 77%, also report high internal demands to be a factor of importance for their current situation at follow‐up, whereas the group that have recovered seem to have manage somewhat better to make changes related to high internal demands. The change regarding emotional demands is also relative larger in the group that have recovered since it was significant between the groups at baseline but not at follow‐up. In a previous study, in the process of return to work, workers with common mental disorders experience decreased working capacity due to mental or physical symptoms such as tiredness and reduced concentration but describe difficulty in protecting themselves from exceeding their current capacity (Noordik et al., 2011). The main barriers related to the difficulty of protecting themselves are setting limits in a demanding situation, recognizing their current capacity related to exhaustion, and to control cognitions such as perfectionism. Thus, patients with OCPD that have clinically recovered from ED might have been able to better deal with these barriers including protecting themselves from exceeding capacity reflected in reduction in emotional demands.

It is also noticeable that as many as 82% of the group with OCPD that have clinically recovered from the exhaustion reported their managerial responsibility to be a contributing factor to their exhaustion at baseline compared to 18% at follow‐up. Thus, it could be speculated that changes in the work situation for managers seems to of importance for recovery, either in form of withdrawal from managerial responsibility or improved working situation resulting in decrease the workload and demands. This needs to be further studies, focusing explicitly on managers and their situation.

Comorbid depression and anxiety is common among patients with ED and as long as they are suffering from ED, some of the patients are also struggling with depression and anxiety. However, anxiety and/or depression do not seem to be a driven mechanism explaining the differences between the ED+OCPD and EDrec+OCPD groups. Somewhat, larger percent of patients in the ED group scored above the cut‐off for depression but this did not reach significant difference due to small sample size.

*Strengths and limitations*. To our knowledge, a longitudinal study like this, following patients with clinical burnout has not been previously conducted, which is a major strength of this study. However, there are several limitations that need to be discussed. The patients were originally referred to a specialist clinic from primary care centers. Since the diagnostic criteria for ED were relatively new at the time, many primary care physicians were uncertain of the criteria and perhaps unaware of the existence of the specialist clinic where the study was conducted. It is likely that the most severe cases were referred to the clinic, and this population might not entirely represent patients with ED in primary care. However, the study group accepting to participate did not differ from nonresponders. Thus, conclusions drawn can plausible be valid for the ED group as whole treated at specialist clinic. Studies are being conducted with the aim of comparing the specialist care unit population with patients treated solely in primary care. One major limitation is the small study sample, particularly when comparing the groups who fulfill the criteria for OCPD. Larger studies should be performed to better analyze factors related to OCPD and recovery of patients with stress‐related exhaustion. Another limitation of the study is that it is not possible to draw conclusions whether OCPD is a causal factor of exhaustion. To draw conclusions about causality, further studies using different research design are needed. Additional limitation is that the patients are asked to retrospectively state the main stressors that contributed to their exhaustion. The categories of stressors included in the questionnaire are, however, familiar to the patients as these are the stressors that patients with ED stated to be contributors to the exhaustion when they first sought care. A further potential limitation might be that only a specific number of questions from the validated instrument SCID‐II was used to ascertain the level of OCPD. It is difficult to determine whether this procedure has affected the reliability and validity of the instrument. The reason for this procedure was to reduce the response burden for the study participants.

*Clinical implications*. The results of this study indicate that factors related to OCPD may be one of many factors of clinical importance for the patient's recovery from ED. Thus, presence of compulsive behaviors could be a complicating factor in the treatment of ED. These behavioral factors need to be attended to during treatment in this subgroup of patients with ED, and these patients should be identified when the initially seek care or as early as possible. A meta‐analysis showed that CBT treatment of perfectionism not only reduced perfectionist symptoms but also reduced symptoms of depression and anxiety (Lloyd et al., 2015; Mahmoodi et al., 2020). Thus, CBT treatment should be considered for this subgroup of patients, but further studies need to be conducted to elucidate if symptoms of exhaustion can be affected by therapeutic interventions in these patients.

## CONFLICT OF INTEREST

None declared.

## Significant Outcomes

- Patients with residual clinical ED report OCPD to a greater extent compared with patients who do not longer fulfill the clinical criteria for ED
- 7–10 years after seeking care.
- The OCPD symptom “Excessive devotion to work” is more common among patients with OCPD who have not recovered from ED compared with patients with OCPD who have recovered. Other OCPD symptoms did not differ between the groups.
- Patients with OCPD who have recovered from ED and patients with OCPD that have not recovered report similar pattern regarding perceived contributing exposure factors at work and/or in private life.

## Limitations

- The patients were originally referred to a specialist clinic from primary care centers. It is possible that the most severe cases were referred to the clinic
- and this population might not entirely represent patients with ED in primary care.
- One major limitation is the small study sample
- particularly when comparing the groups who fulfill the criteria for OCPD. Larger studies should be performed to better analyze factors related to OCPD and recovery of patients with stress‐related exhaustion.
- Another limitation of the study is that it is not possible to draw conclusions whether OCPD is a causal factor of exhaustion. To draw conclusions about causality
- further studies with different research design are needed.
- Only a specific number of questions from the validated instrument SCID‐II have been used to ascertain the level of OCPD. It is difficult to determine whether this procedure has affected the reliability and validity of the instrument. The main reason for this procedure was to reduce the burden for the study participants

### PEER REVIEW

The peer review history for this article is available at https://publons.com/publon/10.1002/brb3.2171.

## Data Availability

The data that support the findings of this study are available from the corresponding author upon reasonable request.

## APPENDIX 1

This questionnaire aims to identify the factors that contributed to the stress leading to your exhaustion, that is, stressors. Check box **A** if you consider this stressor to be a **contributing factor at the time point when you first became ill** with exhaustion. Check box **B** if you consider the factor to be a **significant burden in your life today**. You can check both boxes if relevant.

**Table:**

	A *Then*	B *Now*
**Quantitative demands at work (e.g., high workload, high time pressure)**		
Emotional demands (e.g., work tasks exceeding what you can handle or have competence for, emotional demanding responsibilities for clients/patients/students etc.)		
**Conflicts at work (e.g., with co‐workers, managers, or clients)**		
**Managerial responsibilities (i.e., leadership situations that are perceived as stressful)**		
**Reorganization/changes at work**		
**Deficient leadership (you immediate or senior management)**		
**Job insecurity (e.g., project position or unemployment)**		
**Irregular working hours/shift work**		
**Traumatic event at work (e.g., legal proceedings, threatful situations at work, violence, or threats of violence at work)**		
**Poor physical work environment (e.g. high noise level, presence of chemicals, heavy lifting)**		
**Discontent at work**		
**Death of a family member**		
**Caring for a family member (e.g., because of physical or mental illness)**		
**Being a single parent**		
**Relational conflicts in private life**		
**Separation**		
**Change in family composition**		
**Worries about your health**		
**Worries about personal injury or illness, not connected to the exhaustion**		
**Financial worries**		
**Residential worries**		
**Worries connected to voluntary engagement (overload in voluntary activities)**		
**Legal worries (e.g., legal process or similar processes)**		
**Loneliness**		
**High internal demands on yourself**		
**Existential worries about life and decisions about the future**		
**Other:**		
